# Comparative analysis of the immune response to RFA and cryoablation in a colon cancer mouse model

**DOI:** 10.1038/s41598-022-22279-w

**Published:** 2022-10-29

**Authors:** Michal Mauda-Havakuk, Natalie M. Hawken, Joshua W. Owen, Andrew S. Mikhail, Ankit Saxena, Baktiar Karim, Paul G. Wakim, William F. Pritchard, John W. Karanian, Bradford J. Wood

**Affiliations:** 1 Center for Interventional Oncology, Radiology and Imaging Sciences, Clinical Center, and the National Institute of Biomedical Imaging and Bioengineering, National Institutes of Health, Bethesda, MD USA; 2 Center for Interventional Oncology, Radiology and Imaging Sciences, Clinical Center, National Institutes of Health, Bethesda, MD USA; 3 Flow Cytometry Core, National Heart, Lung, and Blood Institute, Clinical Center, National Institutes of Health, Bethesda, MD USA; 4 National Cancer Institute, National Institutes of Health, Frederick, MD USA; 5 Biostatistics and Clinical Epidemiology Service, Clinical Center, National Institutes of Health, Bethesda, MD USA; 6 Center for Interventional Oncology, Radiology and Imaging Sciences, Clinical Center, National Institutes of Health, 10 Center Drive, Room 3N320B, MSC 1182, Bethesda, MD 20892 USA

**Keywords:** Immunology, Oncology

## Abstract

The immune response to radiofrequency ablation (RFA) and cryoablation (CRA) was characterized and compared in a colon cancer mouse model. All studies were conducted under a research protocol approved by the National Institutes of Health, Clinical Center, Animal Care and Use Committee. BALB/cJ mice were inoculated with CT26 cells, and randomized to RFA, CRA, or sham treatment. Mice were sacrificed 3 days post-treatment, and tumor, spleen, and serum were harvested. Cell death was determined by Caspase-3 immunohistochemical and TUNEL stains. Immune response was analyzed using flow cytometry, serum cytokine assay and immunohistochemistry. Cell death, necrosis, and apoptosis induced by ablation were comparable in RFA and CRA. Decreased frequency of systemic T-regulatory cells was found in the CRA group. Both RFA and CRA reduced frequencies of several myeloid-derived suppressor cell (MDSC) subpopulations. RFA induced pro-inflammatory cytokine secretion including TNF-α and IL-12 as well as anti-inflammatory cytokines IL-5, and IL-10. CRA augmented secretion of a wider array of cytokines compared to RFA with both pro- and anti-inflammatory properties including IL-1β, IL-5, IL-6, IL-10, and KC GRO. In the tumor microenvironment, RFA reduced the number of T-regulatory cells, a finding not observed with CRA. Reduction of immune suppression via decreases in T-regulatory cells and MDSC was found to be induced by RFA or CRA. CRA augmented a wider range of cytokines than RFA, which were mainly pro-inflammatory, but also anti-inflammatory. In the tumor microenvironment, RFA demonstrated more pronounced anti-tumoral immunity. Further delineation of specific immunomodulation induced by ablation could inform drug-device development and may play a role in future hypothesis-driven immunomodulatory paradigms that combine immunotherapy drugs with tumor destruction for the treatment of metastatic colon cancer.

## Introduction

Tumor ablation may play a role in treating certain patients with hepatocellular carcinoma, renal cell carcinoma, or colon cancer^1–3^. Percutaneous ablation may provide minimally invasive and precise tumor eradication with low complication rates^4^, shorter hospital stays^5^, and reduced costs^6^. Among many ablative modalities, the longest-term experience has been gained with radiofrequency ablation (RFA) and cryoablation (CRA). Comparative analysis between ablation modalities is limited, and has focused mainly on assessment of complication rates^7^ or evaluation of ablation margin sharpness^8^. Immunotherapy has revolutionized cancer care, however, there remains a need to increase patient response rates to immunotherapy for solid tumors. One approach to achieve this goal is to harness combinational therapy potential such as ablation and embolization in conjunction with immunotherapy to enhance the immune response. To efficiently and safely combine immunotherapy and ablation treatments, characterization of the immune effects caused by ablation modalities is critical. Ablation eradicates tumor cells in situ and thus has the potential to expose tumor antigens and uncover or release neo-antigens that may stimulate anti-tumoral immune responses. There is substantial interest in utilizing RFA or CRA to potentiate an anti-tumoral immune response, however clinical data that directly compares immune responses among different ablation modalities is scarce. RFA is widely used to treat liver tumors, and its anti-tumoral immune behavior has been characterized as inducing tumor-specific T cells expansion and decreasing the frequency of myeloid-derived suppressor cells (MDSC)^9,10^. Further, in 2 of 6 patients with colorectal liver metastasis, RFA-induced T cell-mediated tumor recognition was shown by stimulation of pre- and post-treatment peripheral blood mononuclear cells (PBMC) with selected peptides eluted from the tumor major histocompatibility complex molecules^11^. CRA is commonly used for treatment of kidney, lung, bone, liver, and soft tissue tumors. Expansion of certain T cell clones in tumor tissues was found post-CRA of kidney tumors^12^ and a decrease in T regulatory cells was found in prostate cancer patients that received CRA^13^. A clinical study investigating the PBMC response to ablation suggested that heat-based ablation (RFA or microwave) augmented immune responses that favor anti-tumoral effects, whereas CRA increased both anti-tumoral and suppressor responses^14^. While this study is suggestive, it did not definitely consider immune infiltrates in the tumor microenvironment (TME) or systemic immune cells.

Pre-clinical data comparing ablation modalities is slightly more abundant but suffers from translational limitations. A study investigating CRA, RFA, and laser ablation found more profound immune activation post CRA^15^ but was limited to evaluation of only a few immune markers, which precluded a more comprehensive analysis. Most importantly, ablations were not conducted in tumors but in normal rat livers. A study with similar limitations showed a more robust immune response post RFA and CRA versus microwave ablation or hepatic resection^16^. More recently it was suggested that a CRA-RFA combination provides superior protection against tumor rechallenge compared to RFA alone in a murine melanoma model^17^. However, this treatment regimen does not recapitulate standard clinical practice.

A comparative analysis of the immune response to RFA or CRA was conducted in a colon cancer tumor model in mice to better elucidate the immune response evoked by different ablation modalities. We performed ablations in line with clinical protocols and ablation endpoints and conducted a comprehensive immune investigation including tumoral, functional, and systemic immune markers post ablation. Mechanistic insight into the ablation immune response may inform new treatment algorithms and guide ablation and immunotherapy combination selection for future study.

## Methods

### Animals and cell line

**Figure 1:**
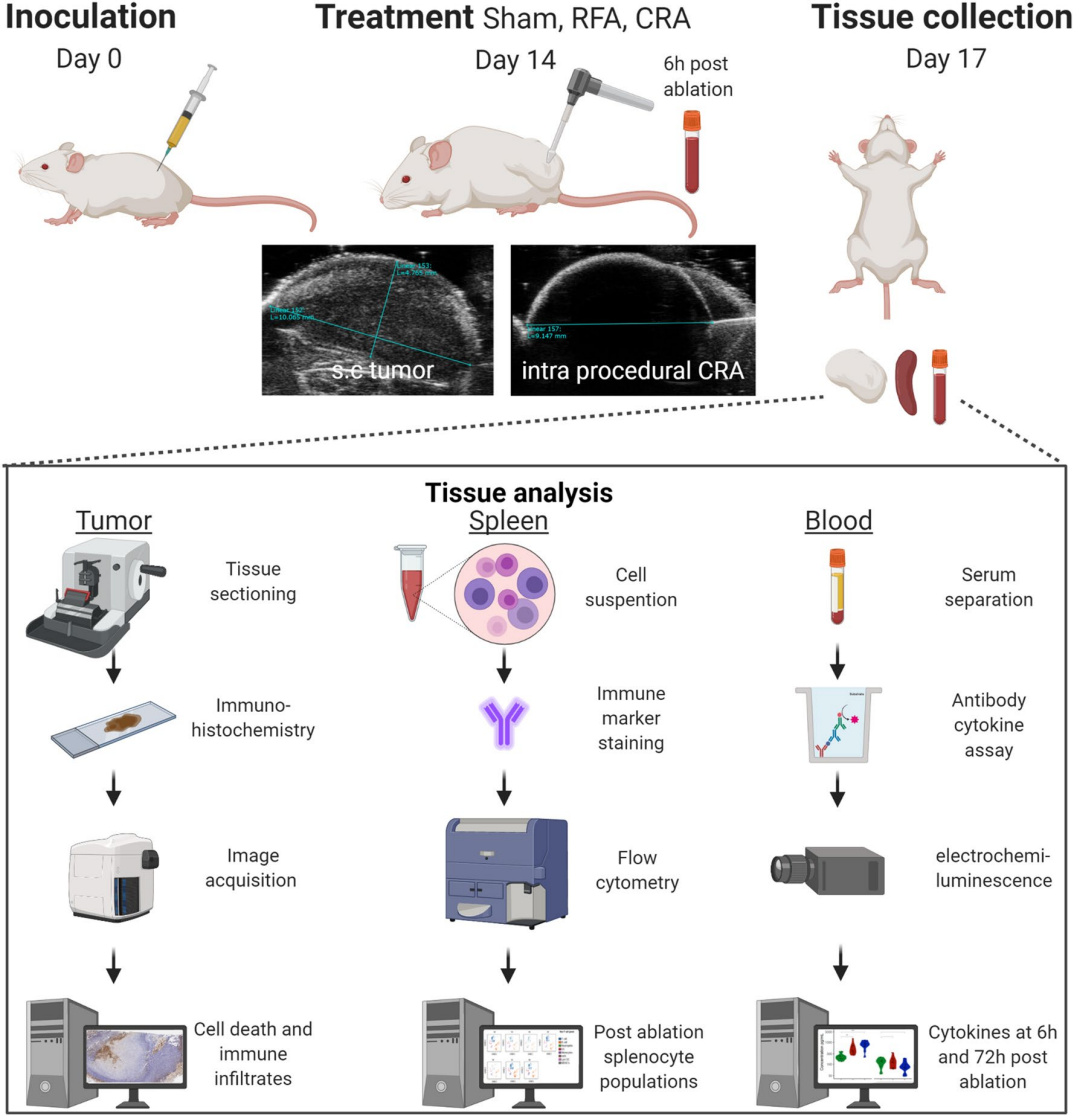
Study design and methods. Mice were subcutaneously inoculated with CT26 colon cancer cells. After 14 days, tumors were treated with sham procedure, radiofrequency ablation (RFA), or cryoablation (CRA) using ultrasound guidance. Three days post ablation, mice were sacrificed, and tumor, spleen, and serum were harvested. Tumors were analyzed by immunohistochemistry for cell death, apoptosis, and immune cell infiltration. Splenocyte cell suspensions were stained with multiple antibodies and expression of various immune cell sub-populations were measured with flow cytometry. Circulating cytokines were measured at two time points: 6 h post ablation blood draw and 3 days post ablation at sacrifice.

This study was conducted under an animal use protocol approved by the National Institutes of Health, Clinical Center, Institutional Animal Care and Use Committee in compliance with the U.S. Animal Welfare Regulations, all methods are reported in accordance with ARRIVE guidelines. Schematic presentation of the study timeline and methods is provided in Fig. 1.

BALB/cJ mice, 6–8 weeks old (Jackson Laboratory, Bar Harbor, ME) were housed and maintained in specific pathogen-free conditions. Colon cancer cell line CT26, kindly provided by Kelly Dougherty/Carrie Bonomi (NCI, Frederick), was maintained by culturing in RPMI-1640 medium (Gibco, Life Technologies, Cat# 11835030) with 10% heat-inactivated fetal bovine serum (HyClone, GE Healthcare Life Sciences Cat#SH3007003HI), 100 U/mL penicillin, 100 μg/mL streptomycin, and 2 mM l-glutamine, and (Gibco, Cat#10378016). Mice were inoculated by subcutaneous flank injection with 2.5 × 10^5^ cells suspended in 100 μL phosphate buffered saline (PBS) pH 7.4 (Quality Biological. Cat#114-058-101) using a 25-gauge hypodermic needle. Flank tumors were monitored and measured with electronic calipers every 3 days. Tumor volumes were calculated according to the following formula: volume = long dimension × short dimension^2^/2. When tumors reached a mean volume of 100 mm^3^, mice were divided randomly into RFA and CRA ablation cohorts (n = 10 each) and a sham control group (n = 6). Hair around the tumor was clipped and removed by depilatory cream before ablations.

### Ablation procedures

Ablation treatment algorithms for RFA and CRA were chosen to replicate clinical protocols with the aim of achieving complete tumor ablation. Mice were placed in an anesthesia induction chamber (4% isoflurane, 500–1000 mL/min oxygen). Once anesthetized eye lubricant was instilled, mice were placed in the prone position, and anesthesia maintained via nose cone (2–3% isoflurane, 100–200 mL/min oxygen). Body temperature was monitored during the procedure using a rectal probe and maintained with heat packs. Intraprocedural ultrasound guidance allowed for precise ablation probe placement along the tumor’s long axis, minimizing the confounding degree of over-ablation or non-target ablation. The insertion site was cleaned with alcohol wipes and the probe was kept in aseptic conditions between procedures. Three treatment protocols were followed: RFA was performed with a 22-gauge probe with a 4 mm uninsulated tip (Baylis Medical, Montreal, QC, Canada) for 1.5 min at 90 °C; CRA was performed with an 18.5-gauge probe (Galil, Biocompatibles, Farnham, UK) using two cycles of 100% freeze intensity of 1 min duration and 0.5-min passive thaw, for a total treatment time of 3 min; and sham procedures were performed by insertion of either the RFA or CRA probes (N = 3 each) without energy or gas, for the same duration as the treatment cohorts. Immediate post procedural analgesia was provided with 0.05 mg/kg SQ buprenorphine. Triple antibiotic ointment was administered (First aid antibiotic/Bacitracin zinc/Neomycin sulfate/Polymyxin B sulfate, Walgreens) on the probe access site, and mice were observed until full recovery and then daily until termination on day 3.

### Tissue harvesting and processing

Six hours after the procedure, blood was collected via retroorbital sinus puncture. Serum was separated and cryopreserved. Three days after the procedure, mice were euthanized by CO_2_ inhalation. Blood was collected via direct cardiac puncture. The spleen, lymph nodes, and tumor were collected. Splenic cells were isolated using a 70 μm cell strainer, washed with cold incomplete RPMI, purified using ACK lysis buffer, counted, and cryopreserved. In preparation for flow cytometry, frozen aliquots of splenic cells were thawed, suspended in cRPMI, and centrifuged (1200 rpm, 4 °C, 7 min). Following resuspension in warm cRPMI, the cells were loaded into 6-well plates and incubated for 24 h (37 °C, 5% CO_2_). Non-adherent cells were transferred to a conical tube, centrifuged and resuspended in cold separation buffer (PBS pH 7.2, 0.5% Bovine Serum Albumin, 2 mM EDTA). Cells were transferred to a V-bottom tissue plate, stained with blue dead cell stain (Invitrogen, cat#L34962) for 30 min on ice in the dark, and blocked with CD16/CD32 (Mouse BD Fc Block™—BD Biosciences, San Jose CA, USA Cat#55314). Cells were incubated with antibodies in two different panels for 45 min on ice in the dark using a 5% mixture of normal rat and normal mouse serum in FACS buffer for blocking. Intracellular and intranuclear marker stains were performed following incubation of cells with permeabilization buffer (eBioscience™ Protein Transport Inhibitor Cocktail cat# 00-4980-03 and FOXP3 Transcription Factor Staining Buffer Set cat# 00-5323-00 respectively). After surface staining, the cells were fixed, permeabilized, and stained for interferon gamma (INF-γ), CD8a, tumor necrosis factor-α (TNF-α), IL-17a, FOXP3, interleukin-2 (IL-2), interleukin-4 (IL-4). The complete antibody list is provided in Supplementary Table S1. Subsequently cells were washed, fixed, and analyzed by flow cytometry (FACS Symphony, 4 lasers, BD Biosciences). Unstained cells and compensation beads (UltraComp eBeads, Invitrogen cat#01222242) served as controls. Data analysis was performed using FlowJo software (MAC-Version 9.8.6., BD Biosciences).

### Clustering analysis and t-SNE visualization

Clustering analysis was performed to analyze and visualize the high-dimensional nature of the flow cytometry data. First, FSC-A/SSC-A was applied and gated for Live/Dead cells. Then, T cell or non-T cell marker gates were applied. Fifty-thousand cells were randomly sampled from the final gate per animal for our analysis and a t-distributed stochastic neighbor embedding (t-SNE) Barnes-hut algorithm was used to reduce the dimensionality of the data set for visualization.

### Immunohistochemistry (IHC) and TUNEL stains analysis

Tumors tissues were fixed in formalin for a minimum of 24 h and processed through a series of increasing concentrations of ethanol, cleared in xylenes, and infiltrated with paraffin wax. Tissues were then embedded in paraffin blocks and sectioned. Sequential 5 μm thick sections from the paraffin blocks were mounted onto glass slides (Fisher Scientific, Waltham, MA, USA). The use of sequential adjacent tumor sections enabled spatial histological correlation of stains, For histologic examination and immune marker immunohistochemical analysis, consecutive tissue sections were obtained from tumors and were independently stained with hematoxylin and eosin (H&E), terminal deoxynucleotidyl transferase dUTP nick end labeling (TUNEL) (ApopTag Peroxidase In-Situ Apoptosis Detection Kit, cat#S7100, Millipore, Burlington, MA, USA), and cleaved caspase-3 (CASP3) (Cell Signaling Technology cat#9661). The cell death region was first evaluated on H&E in which cell death is illustrated as the absence of hematoxylin (stained as dark purple) and the presence of eosin (stained as pink). Sections were also stained for immune infiltrates. IHC automated staining was performed on LeicaBiosystems’ BondRX with the following conditions: Bond Epitope Retrieval 1 (Citrate) 20ʹ for CD3 (Bio-Rad #MCA1477 rat monoclonal, 1:100 incubated 60ʹ), CD8a (eBioscience #14-0195-82 rat monoclonal, 1:50 incubated 30ʹ), and Cleaved Caspase 3 (Cell Signaling Technology #9661, 1:100 incubated 60ʹ), whereas Proteinase K (DAKO/Agilent #S3020 5ʹ room temperature) was used for F4/80 (eBioscience #14-4801 rat monoclonal, 1:200 incubated 60ʹ). The Bond Polymer Refine Detection Kit (LeicaBiosystems #DS9800) with omission of the PostPrimary Reagent was used, and a mouse adsorbed rabbit anti-rat secondary antibody (Vector Labs) was included for CD3, CD8a, and F4/80. Normal mouse spleen served as positive control tissue for CD3, CD8a, and F4/80 whereas normal mouse thymus was used for Cleaved Caspase 3. Isotype control reagents were used in place of the primary antibodies for the negative controls.

Manual staining was performed for FOXP3 using the avidin–biotin IHC method. Following antigen retrieval with citrate buffer at pressure with Biocare’s Decloaking Chamber, slides were incubated 60ʹ with FOXP3 (eBioscience #14-5773 rat monoclonal, 1:100), followed by a mouse adsorbed biotinylated rabbit anti-rat secondary antibody (Vector Labs), ABC Elite (Vector Labs), DAB was the chromogen, and hematoxylin the counterstain.

Normal mouse spleen served as positive control tissue. Isotype control reagent was used in place of the primary antibody for the negative controls. H&E slides, TUNEL slides, and IHC stained slides were scanned at 20× using an Aperio AT2 scanner (Leica Biosystems, Buffalo Grove, IL) into whole slide digital images. All image analysis was performed using HALO imaging analysis software (Indica Labs, Corrales, NM). Slides were then thoroughly annotated by a pathologist (BK) who was blinded to the status of the groups of mice. The tumor and surrounding margins (100 μm) were manually annotated. Artifact such as folds, and tears were excluded form analysis. Image analysis was performed on one section using the cytonuclear algorithm in HALO version 3.2 to determine percent positive cells.

### Serum cytokine assay

An electrochemiluminescence assay (V-PLEX Proinflammatory Panel 1 Mouse Kit K15048D-1, Meso Scale Diagnostics, LLC, Gaithersburg, MD, USA) was used for detection of the following cytokines according to the manufacturer’s instructions: IFNγ, TNFα, interleukin 1β (IL-1β), IL-2, IL-4, interleukin-5 (IL-5), interleukin-6 (IL-6), interleukin-10 (IL-10), interleukin-12p70 (IL-12p70), keratinocyte chemoattractant growth-regulated oncogene (KC/GRO). Briefly, antibody solutions were added at room temperature, incubated and washed as per manufacturers protocol. The 96-well plate was immediately analyzed using a plate reader (Meso Sector S 600, Meso Scale Diagnostics).

### Statistical analysis

Statistical analyses were performed using GraphPad Prism version 9.0.0 (GraphPad Software, San Diego, CA, USA). An ordinary one-way ANOVA with Tukey’s multiple comparisons test and single pooled variance was performed. For scatter dot plots, the mean is represented by a scattered dot box and whiskers reflect the standard error of the mean. A repeated-measures model was used to account for the correlation between cytokine levels within the same animal where the 6 h measure, and 72 h measure were kept separate. The dependent variable was defined as cytokine level, and independent variables were (1) treatment: Sham, RFA, CRA; (2) time: 6 h vs. 72 h; and (3) interaction between (1) and (2). The cytokine levels between different pairs of groups were compared based on t-tests applied to differences in least-squares means obtained from the model. Because all reported p-values are unadjusted for multiple comparisons, and using the general Bonferroni rule of thumb, a p-value less than 0.016 (≈ 0.05/3 tests) was used as a target threshold of possible relative differences. The repeated-measures analyses were performed using SAS Version 9.4 software (SAS Institute, Inc., Cary, NC, USA). Graphs were created using RStudio software (Version 1.2.5033, Integrated Development for R, RStudio, PBC, Boston, MA, USA). Violin graphs were used to visually display a kernel density estimate and individual samples were represented as dots.

## Results

### RFA and CRA ablation regions were comparable in size and type

**Figure 2:**
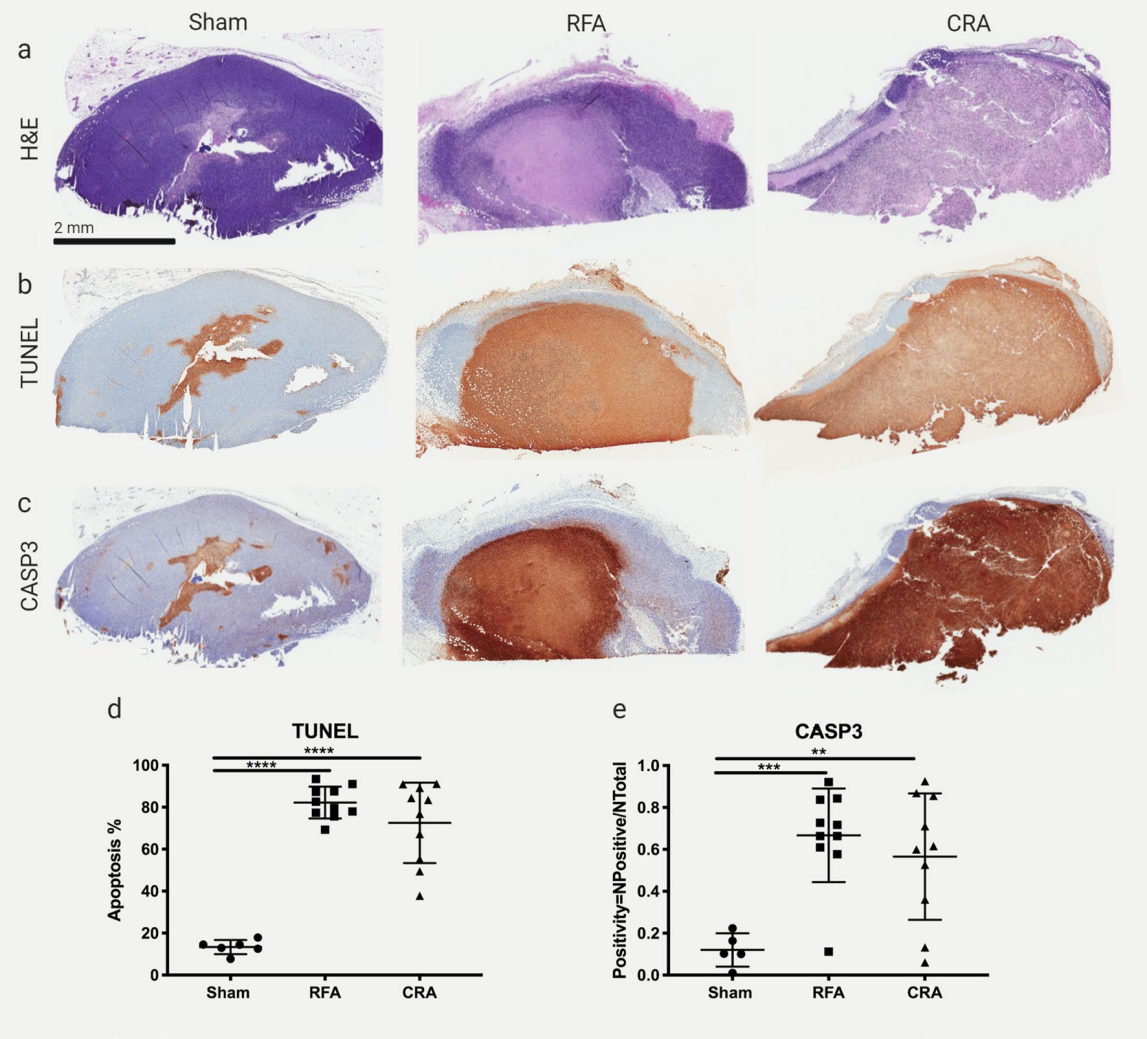
Immunohistochemical analysis of cell death. (**a**) Representative H&E-stained tumor sections from sham, RFA, and CRA treatment groups. (**b**) TUNEL and (**c**) Caspase-3 (CASP3) stains of tumor sections corresponding to the H&E sections. (**d**) TUNEL and (**e**) CASP3 stain quantification. Mean and SD are denoted by large and small bars, respectively. Ordinary one-way ANOVA was performed. **p < 0.01, ****p < 0.0001. Statistical analyses were performed using GraphPad Prism version 9.0.0.

Sham procedures caused minimal cell necrosis, mainly along the needle track. RFA and CRA treated tumors had large regions of coagulative necrosis that encompassed most of the tumor. H&E stains are shown in Fig. 2a. The presence of DNA fragmentation (TUNEL stain, Fig. 2b) overlapped with the CASP3 stain (Fig. 2c) for both RFA and CRA. For characterization of the type of cell death, CASP3 stain was performed. CASP3 is an apoptotic cell marker and cells that underwent apoptosis are stained brown while all other cells are unstained. The degree of DNA fragmentation was significantly higher in RFA and CRA treatments (82% and 72% respectively) compared to sham (13%) treatment and no difference was found between RFA and CRA (Fig. 2d). CASP3 positivity was found to be significantly higher in RFA and CRA (68% and 65% respectively) compared to sham treatment (12%) and no difference was found between RFA and CRA (Fig. 2e).

### RFA and CRA decrease suppressor cell frequencies

**Figure 3:**
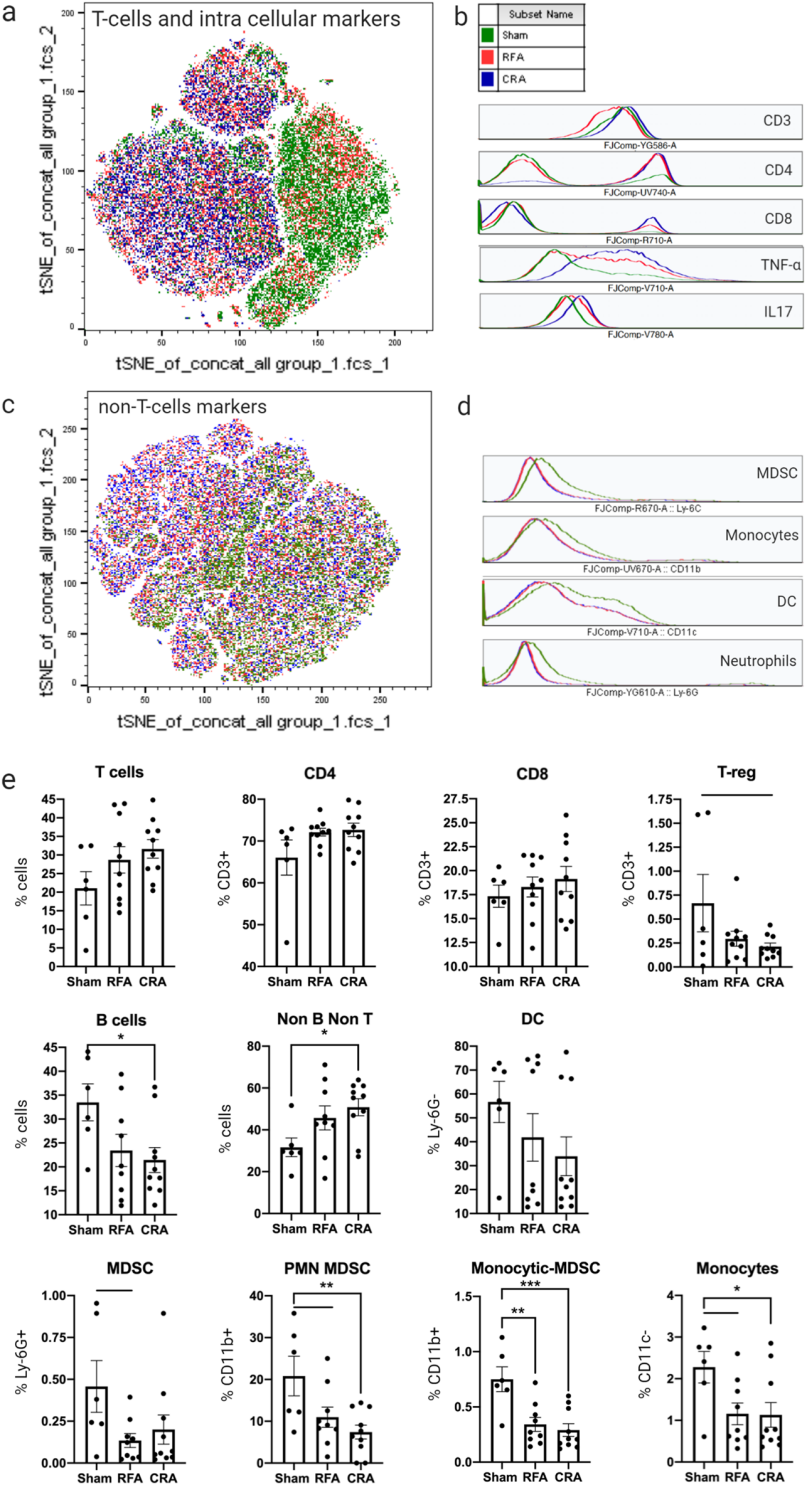
Flow cytometry analysis of splenocytes post ablation. (**a**) t-SNE plot of T-cells markers and (**b**) corresponding histograms. (**c**) t-SNE plot of non-T cell markers and (**d**) corresponding histograms. Treatment groups: sham (green), RFA (red), and CRA (blue). (**e**) Frequency of total T cells (CD3^+^), CD4^+^, CD8^+^, CD4^+^ T regulatory (CD4^+^FOXP3^+^), B (CD3^−^CD19^+^), non-B non-T (CD3-^−^CD19), DC (CD11c^+^), MDSC (CD11b^+^Ly6G^+^), PMN MDSC (CD11b^+^Ly6C^+^), Monocytic MDSC (CD11b + Ly6G^−^ + Ly6C^+^), and monocytes (CD11c^−^Ly6C^+^). Ordinary one-way ANOVA. The mean is represented by the box and whiskers reflect the standard error of the mean, *p < 0.05, **p < 0.01, ***p < 0.001. Group comparison without an asterisk represents a trend at p ≤ 0.07. Statistical analyses were performed using GraphPad Prism version 9.0.0.

The effect of ablation on immune cells was evaluated to better characterize underlying immune alterations for clues to potential mechanisms. Splenocytes served as an approximation of systemic changes and thus were analyzed with flow cytometry (gating strategies are provided in Supplementary Figs. S1, S2). Frequencies of total T cells, CD4^+^, CD8^+^, T regulatory, T naïve, T effector, T central memory, T effector memory, B cells, non-B non-T lymphocytes, dendritic, myeloid-derived suppressor cells, polymorphonuclear neutrophils myeloid-derived suppressor cells, monocytic myeloid-derived suppressor cells, and monocytes, were determined. t-SNE analysis indicated that, for the most part, RFA and CRA groups overlapped and showed the same trends in immune cell frequency changes, and both demonstrated different frequencies from the sham group (Fig. 3a–d). Frequencies of CD3^+^, and CD8^+^ T-cells as well as their subpopulations did not differ between treatment groups (Fig. 3b,e). Higher frequency of CD4^+^ was found in both treatment groups compared to sham (Fig. 3b). In CRA, a trend of lower T-regulatory frequency was noted (p = 0.07). Higher frequency of the heterogenous subpopulation non-B non-T lymphocytes was observed in the CRA treatment which were further characterized and subdivided to specify affected cells. Several sub-populations among the non-B non-T lymphocytes were identified as myeloid derived suppressor cells (MDSC), including polymorphonuclear-MDSC, monocytic-MDSC cells and monocytes, and all were found in lower frequencies in RFA and CRA treatments compare to sham (Fig. 3d,e). This may suggest that another subpopulation accounts for the increase in non-B non-T lymphocytes. CRA treatment also decreased B cells frequency (Fig. 3e).

### RFA and CRA promote secretion of multiple cytokines

**Figure 4:**
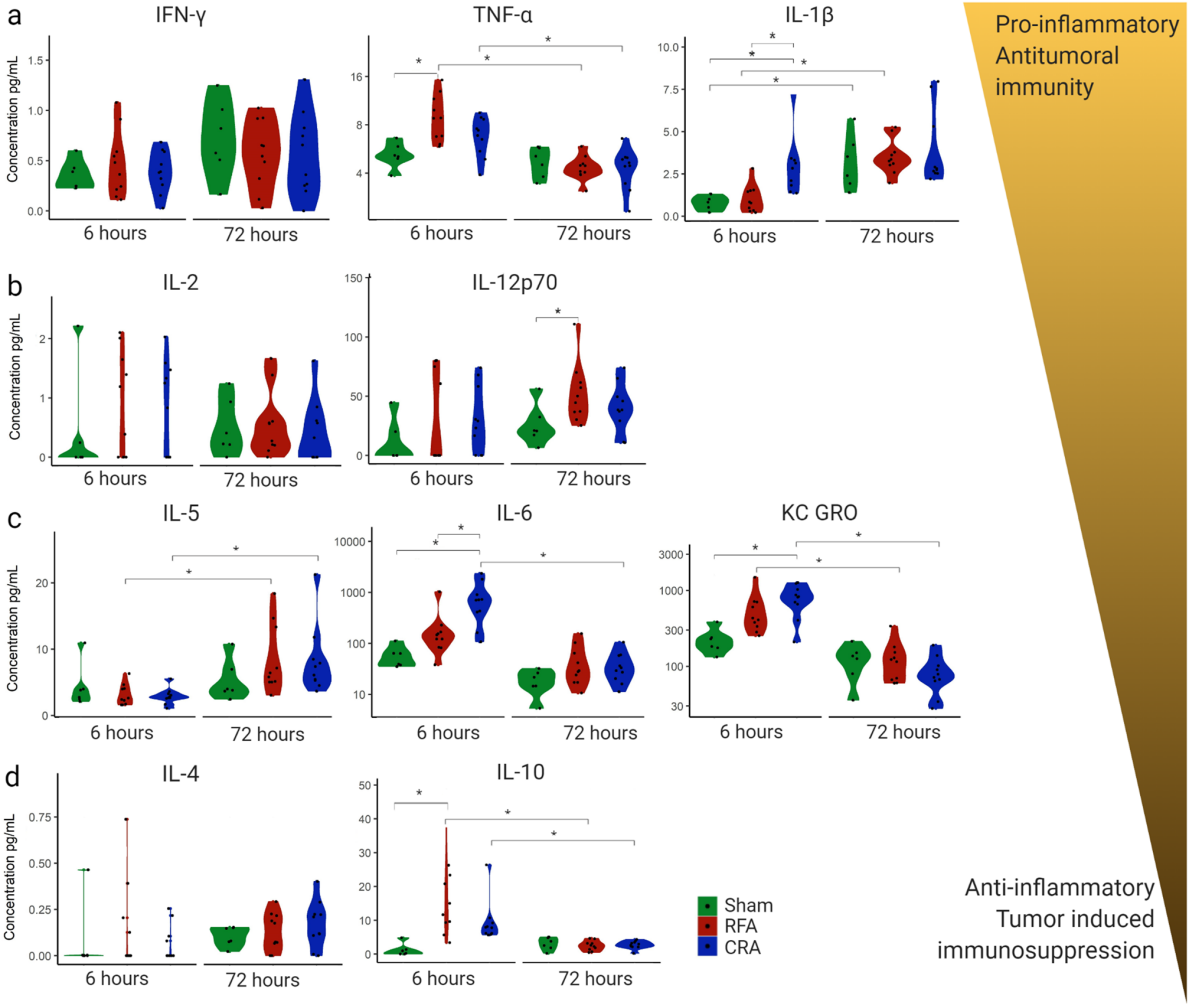
Cytokine secretion 6 and 72 h post ablation. Cytokine secretion in the serum of mice post treatment with sham, RFA, or CRA was measured at 6 h and 72 h post ablation. Cytokines associated with more anti-tumoral response are represented on the top while cytokine with more immunosuppression effects are represented at the bottom. From top to bottom, the secretion of the following cytokines is presented: (**a**) IFN-Ꝩ, TNF-α, and IL-1β, (**b**) IL-2, and IL-12, (**c**) IL-5, IL-6, and KC-GRO, (**d**) IL-4, and IL-10. Repeated-measures model was used to account for the correlation between cytokines level within the same animal as described in methods, *p < 0.016. See Supplementary Tables S2 and S3 for differences in least-squares means and corresponding p-values. The repeated-measures analyses were performed using SAS Version 9.4 software.

To characterize immune cell function post ablation, cytokine secretion was measured in serum at 6 and 72 h. Cytokines play an important role in the inflammatory process and antitumoral immunity. Cytokines can be generally divided into pro- and anti-inflammatory characteristics, despite the fact that each cytokine encompasses both capabilities^18^. Cytokine classification as having either anti-tumoral or anti-inflammatory tumor induced immunosuppressive effects are summarized in Fig. 4. Two cytokines, TNF-α and IL-1β, initiate the immune cascade. TNF-α was induced by RFA treatment, and IL-1β by CRA treatment, at 6 h (Fig. 4a). TNF-α decreased at 72 h and was comparable in all groups but IL-1β elevation persisted at 72 h post CRA although not different than sham or RFA at this time point. Only RFA resulted in elevated pro-inflammatory cytokine IL-12 secretion compared to the sham procedure at 72 h post ablation (Fig. 4b). CRA augmented secretion of KC-GRO, a neutrophil chemoattractant that also plays a role in angiogenesis, at 6 h, its secretion was reduced and comparable with the other groups at 72 h (Fig. 4c). CRA induced secretion of IL-6, and RFA induced IL-10 secretion, at 6 h, and levels of both cytokines diminished and were not different than controls at 72 h. Both IL-6 and IL-10 have dual pro and anti-inflammatory effects. In general, secretion of 5 cytokines (TNF-α, IL-1β, KC-GRO, IL-6, IL-10) were elevated at 6 h post RFA or CRA ablation compared to the 72 h time point in which higher concentrations than control was found only for IL-12 in the RFA treatment group.

### RFA induces anti-tumoral immunity in the tumor microenvironment

**Figure 5:**
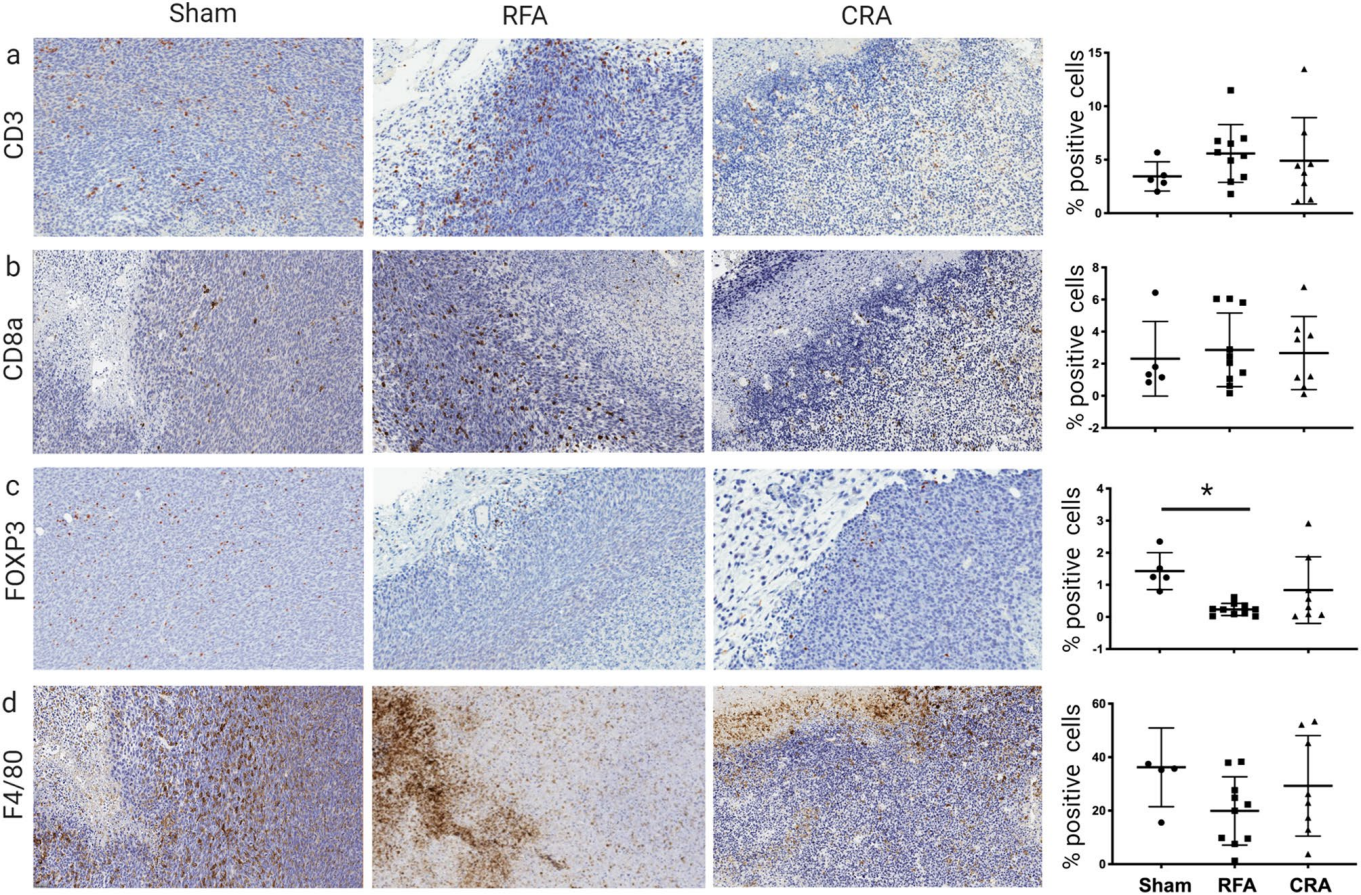
Tumor microenvironment immune infiltrates post ablation. Representative immunohistochemical (IHC) expression of immune infiltrates for sham, radiofrequency ablation (RFA) and cryoablation (CRA) treatment groups: (**a**) CD3, (**b**) CD8a, (**c**) FOXP3 (T-reg), and (**d**) F4/80 (macrophages). The column on the far right represents IHC marker expression levels for each treatment group. Ordinary one-way ANOVA, line at mean with SD, *p < 0.05. Inter-group comparison without an asterisk represents a trend at p = 0.078. Statistical analyses were performed using GraphPad Prism version 9.0.0.

The immune milieu of the tumor microenvironment (TME) was analyzed as a measure for anti-tumoral immunity. IHC analysis was chosen over extraction of tumor infiltrating lymphocytes, to preserve immune cell spatial distribution. Staining and quantification of immune cells in the post ablation TME included the entire tumor, ablation zone and its surrounding margin (100 μm) in all samples. Most immune cells were found to reside in the periphery of the ablation zone (Fig. 5). In a quantitative analysis no differences in CD3^+^, CD8 a^+^, or macrophages (denoted as “F4/80”) were found among the different groups (Fig. 5a,b,d) however RFA treatment was shown to reduce T regulatory cells counts (denoted as “FOXP3” Fig. 5c).

## Discussion

An ablation modality that optimally augments anti-tumoral immune responses has not been specifically identified and the specific immune response mechanisms induced by ablation remain elusive. In this study, the immune responses to RFA and CRA were evaluated in a colon cancer murine model. We found that both RFA and CRA reduced immune suppressive effects. CRA induced a wider range of cytokine secretion than RFA, with both anti-tumoral and anti-inflammatory profiles. In the TME, RFA demonstrated more pronounced anti-tumoral immunity, by the limited definitions and profiles studied here. Distinct alterations in cell populations were seen in flow cytometry, IHC tumor micro-environment immune cells, and cytokines in subjects treated with RFA and CRA.

Reduction of immune suppression was found after either RFA or CRA. Both RFA and CRA reduced MDSC frequency in the systemic circulation. Ablation induced a reduction in the total MDSC count including polymorphonuclear, and monocytic cell subpopulations. MDSC are a heterogenous population of immature myeloid cells with potential immune suppressive activity. Studies have shown these cells are not only essential in the regulation of inflammatory events, but also play a role in driving various crucial processes in tumorigenesis including angiogenesis^19^. The presence of MDSC in cancer patients and their tumor promoting functions are well appreciated^20^. In addition, CRA was associated with a reduction in T-regulatory cells in the systemic circulation, whereas RFA reduced T-regulatory cell accumulation in the TME. The regulatory linkage between MDSC and T-regulatory cells remains unclear, but it is possible that MDSC are involved in T-regulatory cell differentiation and that both subpopulations are linked in a common immunoregulatory network^20^.

The immune response is comprised of both anti-tumoral and anti-inflammatory signals. Measuring an array of cytokines enabled insightful examination of their interactions. We found that RFA induced cytokine secretion suggestive of an anti-tumoral effect while CRA induced both anti-tumoral and anti-inflammatory cytokines. This observation is consistent with results of a clinical trial that measured the anti-tumoral and suppressor T-cell ratio in the systemic circulation after either hot or cold ablation and suggested that heat-based ablations offer more anti-tumoral effects compared to CRA^14^. In our work, a correlation between systemic immune effects and immune cells in the TME was made. The anti-tumoral effects after RFA treatment were reproduced in the TME in the form of fewer T-regulatory cell infiltrates. RFA induced IL-10 secretion, but not IL-2, and IL-4. IL-10 inhibits dendritic cell differentiation from monocytes, promotes the differentiation to mature macrophages and inhibits antigen presentation while it stimulates endocytic activity^21^. Others have shown that IL‐10 can stimulate the anticancer activity of intratumoral innate immune effectors, such as NK cells^22^. In turn dendritic cells and tumor‐specific T cell adaptive immune responses might be promoted^23^. Due to their reduced frequency in our results, T-regulatory cells might not contribute to the observed IL-10 secretion. The slight increase in CD4^+^ T cells concomitant with the decrease in T-regulatory cells may suggest that IL-10 is secreted by Tr1 cells^24^. Tr1 cells have been shown to mediate the killing of tumor-promoting macrophages through the secretion of granzyme B and perforin^25^.

Analysis of temporal expression of cytokines also provides an opportunity to propose pharmaceutical interventions that may be used to optimize anti-tumoral immunity via mitigation or manipulation of cytokine disturbances. Certain anti-tumoral cytokines were secreted hours post ablation and diminished rapidly, suggesting the administration of checkpoint inhibitors or other immune modulators may be effective tools in combination with ablation. The current observation that CRA leads to greater anti-inflammatory cytokine secretion than RFA may argue for the combination of anti-rheumatic or anti-neoplastic drugs with CRA. Although speculative, these drugs could potentially tip the scale towards more anti-tumoral cytokine secretion post CRA as they can potentially mitigate cytokines with anti-inflammatory effects while preserving the effects of anti-tumoral cytokines.

The induction of immunogenic cell death (ICD) by ablation may provide a vaccination effect, in which destruction of tumor cells leads to production and secretion of tumor-specific antigens. Eradication of tumor cells that have survived or were missed by ablation may be mediated by an immune cascade in which antigens are taken up by dendritic cells in the presence of robust immunostimulatory cytokines such as interferon type 1, leading to the priming of a T cell adaptive immune response^26^, which may occur across a broad variety of tumor antigens or neo-antigens with broadening of the T-cell receptor repertoire. The degree of tumor destruction, i.e., complete versus incomplete ablation, can also influence the immune response to ablation. Incomplete RFA may provoke a suppressor immune response both in human and pre-clinical models^27^. In our experiments, complete tumor ablation was performed in both treatment groups for several reasons. First, incomplete ablation may lead to an uneven degree of cell death between treatment groups with different levels of antigen and immune stimulation, and thus introduce a bias that might distract from the central question. Second, complete tumor ablation is consistent with most clinical practices with standard indications, and therefore may better frame a purer mechanistic question.

The type of cell death induced by ablation was characterized, given its potential to evoke different death pathways and different levels of immunogenicity and ICD. Although an oversimplified generalization, some consider apoptosis to be more physiological, regulated, and non-immunogenic, as compared to necrosis, which is considered more pathological, incontrollable, and possibly more immunogenic. It has also been postulated that CRA produces necrosis and apoptosis in an immunogenic fashion, whereas other ablation modalities provoke apoptosis with lower immunogenic effects^28^. Our study evaluated the type of cell death induced by ablation in a controlled environment and in a way which allowed comparison between standardized ablation modalities. TUNEL labels cells dying by all forms of regulated necrosis^29^ while CASP3 delineates a more specific apoptotic cell death. We saw no difference in either TUNEL or CASP3 stain between RFA and CRA groups, suggesting some similarity or common pathways of cell death. However, it has become evident that classifying cell death in a dichotomic manner may not be accurate. Regulated forms of necrosis may also participate in tissue homeostasis, and apoptotic cells can also trigger an antigen-specific immune response^30^. Our data suggests that both necrosis and apoptosis are triggered and can even overlap in tumor tissues after RFA or CRA. Cell death morphology and biochemistry as depicted by TUNEL and CASP3 stains may not predict the capacity to provoke ICD. To further delineate, we have investigated the ultimate ICD outcomes: T cell proliferation and commitment. Following antigen recognition, naïve T cells proliferate and differentiate into multiple subsets of memory T cells^31^. RFA and CRA treatments increased CD4^+^ frequency but most of the investigated T cell subsets (CD8^+^ naïve, central memory, effector memory, and effector T cells) did not differ between treatment groups. T-regulatory cells were found in fewer quantities in the TME and in the circulation following RFA and CRA, respectively. Reduction of immune suppression mechanisms, including both anti- and pro-inflammatory cytokine secretion, without notable differences in T cells frequencies may suggest that ablation alone promoted a mild degree of ICD. The observation that RFA and not CRA promoted anti-tumoral effects in the TME may not provide adequate justification to guide ablation modality selection. Until more thorough elucidation of the weighted immunomodulatory mechanisms of each ablation modality, ablation modality selection will likely be determined by practical clinical indications and patient safety, complete tumor eradication, nearby anatomy, organ of tumor burden, local experience, and modality availability.

Our study had several limitations. T cell frequencies were investigated on splenocytes which provide a large quantity of immune cells compared to tumor-draining lymph nodes. However, it is possible that a more profound anti-tumoral immune response could have been detected after ablation via an analysis of nearby draining lymphoid tissue. Tumor-specific immunity with functional assays against tumor associated antigens was not analyzed in this report. We investigated the immune response at 6 and 72 h after ablation. More frequent or prolonged tissue sampling might provide better temporal information on the dynamics of immune response. In fact, it has been suggested that the timing of assay post ablation may be a powerful determinant of cytokine and immune profiles^32^, so a longer time frame could provide better understanding of the adaptive immunity. Although ablation of the entire tumor without adjacent normal tissue damage was the treatment plan, viable tumor cells were inadvertently left behind as shown in Fig. 2. Damage to the skin was avoided during ablations in this subcutaneous tumor model in order to minimize introducing unknown and unforeseen variabilities. This level of care during ablation may have contributed to viable tumor cells remaining. Subtotal incomplete ablation in patients with colorectal cancer may upregulate immune suppression genes or induce immunosuppressive myeloid cell accumulation in residual tumor tissue^27^. Our report provides comparative information on the broad immune responses induced by RFA or CRA in this colon cancer cell line setting under conditions described. The CT26 in-vivo model, unlike human colon cancer, is highly immunogenic^33^, and thus immune response to ablation in this setup might not resemble the expected immune response to ablation in patients. Further investigation in less immunogenic cell lines may provide better insight. Nonetheless, this multicompartmental immune analysis and the characterization of key immune effects could pave the way to ablation and immune modulator combination protocols with the prospect of improving the modest response rates following many immunotherapies.

## Conclusions

Systemic anti-tumoral immune effects in the form of MDSC reduction was more apparent after CRA than after RFA. However, RFA led to generally-associated anti-tumoral cytokine secretion while CRA secreted both generally-associated anti-tumoral and anti-inflammatory cytokines. In the TME, only RFA induced a decrease in T-regulatory cells. These findings may enhance our understanding of the immune effects and potential immune augmentation induced by ablation, which may, in turn, inform future studies of drug-device combination therapies.

## Supplementary Information

Supplementary Information.

## Data Availability

The authors confirm that the data supporting the findings of this study are available within the article and its Supplementary Materials. For additional supporting materials please contact corresponding author.

## Abbreviations

RFA: Radiofrequency ablation
CRA: Cryoablation
MDSC: Myeloid-derived suppressor cells
CASP3: Cleaved caspase-3
PBMC: Peripheral blood mononuclear cells
TME: Tumor microenvironment
ICD: Immunogenic cell death
(H&E): Hematoxylin and eosin
