# Supplementary material for: Comparative analysis of the immune response to RFA and cryoablation in a colon cancer mouse model
Source: Sci Rep. 2022 Oct 29;12:18229. doi: 10.1038/s41598-022-22279-w (PMC9617942; doi:10.1038/s41598-022-22279-w)
Supplement: Supplementary file 1 — Supplementary Information. [file 41598_2022_22279_MOESM1_ESM.docx]

Table S1. Antibodies for flow cytometry analysis.

| **Antigen** | **Dye** | **Isotope and reactivity** | **Clone** | **Cat#** |
| --- | --- | --- | --- | --- |
| CD25 (IL-2 Receptor α) | BB515 | Rat Anti-Mouse | PC61 | BD cat# 564424 |
| IL-17A | BV786 | Rat Anti-Mouse | TC11-18H10 | BD cat# 564171 |
| FOXP3 | PE | Rat anti-Mouse | MF23 | BD cat# 560408 |
| TNF | BV711 | Rat Anti-Mouse | MP6-XT22 | BD cat# 563944 |
| IL-2 | PE-CF594 | Rat Anti-Mouse | JES6-5H4 | BD cat# 562483 |
| IL-4 | PE-Cy7 | Rat Anti-Mouse | 11B11 | BD cat# 560699 |
| IFN-γ | APC | Rat Anti-Mouse | XMG1.2 | BD cat# 554413 |
| CD4 | BUV737 | Rat Anti-Mouse | GK1.5 | BD cat# 612844 |
| CD8a | APC-R700 | Rat Anti-Mouse | 53-6.7 | BD cat# 564983 |
| CD3 | BUV563 | Rat Anti-Mouse | 17A2 | BD cat# 741319 |
| CD107a | PE-Cy^TM^7 | Rat Anti-Mouse | 1D4B | BD cat# 560647 |
| Ly-6C | APC | Rat Anti-Mouse | AL-21 | BD cat# 560595 |
| Ly-6G | PE-CF594 | Rat Anti-Mouse | 1A8 | BD cat# 562700 |
| CD25 (IL-2 Receptor α) | BB515 | Rat Anti-Mouse | PC61 | BD cat# 564424 |
| CD62L (L-Selectin) | BV650 | Rat Anti-Mouse | MEL-14 | BD cat# 564108 |
| CD44 | PE-Cy7 | Rat Anti-Mouse | IM7 | BD cat# 560569 |
| CD11c | BV711 | Hamster Anti-Mouse | HL3 | BD cat# 563048 |
| CD11b (Mac-1) | BUV661 | Rat Anti-Mouse | M1/70 | BD cat# 612977 |
| F4/80 | BV480 | Rat Anti-Mouse | T45-2342 | BD cat# 565635 |
| CD19 | BV605 | Rat Anti-Mouse | 1D3 | BD cat# 563148 |
| I-A/I-E | BUV395 | Rat Anti-Mouse | 2G9 | BD cat# 743876 |

Table S2. Cytokine level differences between treatments at one time point.

| **Time** | **Cytokine** | **Treatment** | **Treatment** | **Estimate** | **95% CI**  **(lower, upper)** | **P value*** |
| --- | --- | --- | --- | --- | --- | --- |
| **6h** | **INF-γ** | Sham | RFA | -0.005 | -0.25, 0.24 | 0.9642 |
|  |  | Sham | CRA | -0.1 | -0.36, 0.14 | 0.3598 |
|  |  | RFA | CRA | -0.1 | -0.3, 0.1 | 0.3185 |
|  | **TNF-α** | **Sham** | **RFA** | **4.3** | **1.9, 6.7** | **0.0013** |
|  |  | Sham | CRA | 1.7 | -0.7, 4.1 | 0.1586 |
|  |  | RFA | CRA | -2.6 | -4.7, -0.5 | 0.0178 |
|  | **IL-1β** | Sham | RFA | 0.4 | -1.2, 2 | 0.6128 |
|  |  | **Sham** | **CRA** | **2.8** | **1.2, 4.4** | **0.0012** |
|  |  | **RFA** | **CRA** | **2.4** | **1, 3.8** | **0.0012** |
|  | **IL-2** | Sham | RFA | 0.5 | -0.2, 1.2 | 0.1873 |
|  |  | Sham | CRA | 0.4 | -0.25, 1.13 | 0.2093 |
|  |  | RFA | CRA | -0.02 | -0.6, 0.58 | 0.9408 |
|  | **IL-12p70** | Sham | RFA | 19 | -13, 51 | 0.2392 |
|  |  | Sham | CRA | 20 | -11, 53 | 0.1953 |
|  |  | RFA | CRA | 2 | -26, 30 | 0.8859 |
|  | **IL-5** | Sham | RFA | -1.5 | -3.6, 0.5 | 0.1385 |
|  |  | Sham | CRA | -1.5 | -3.5, 0.6 | 0.1501 |
|  |  | RFA | CRA | 0.04 | -1.7, 1.8 | 0.9571 |
|  | **IL-6** | Sham | RFA | 238 | -252, 729 | 0.3270 |
|  |  | **Sham** | **CRA** | **819** | **328, 1310** | **0.0020** |
|  |  | **RFA** | **CRA** | **580** | **155, 1005** | **0.0094** |
|  | **IL-4** | Sham | RFA | 0.08 | -0.06, 0.23 | 0.2596 |
|  |  | Sham | CRA | -0.007 | -0.15, 0.14 | 0.9276 |
|  |  | RFA | CRA | -0.09 | -0.2, 0.037 | 0.1614 |
|  | **KC GRO** | Sham | RFA | 321 | -4, 647 | 0.0526 |
|  |  | **Sham** | **CRA** | **555** | **230, 880** | **0.0017** |
|  |  | RFA | CRA | 233 | -47, 514 | 0.0985 |
|  | **IL-10** | **Sham** | **RFA** | **16** | **7.5, 24** | **0.0006** |
|  |  | Sham | CRA | 9 | 1, 11 | 0.0309 |
|  |  | RFA | CRA | -6.7 | -14, 0.5 | 0.0652 |
| **72h** | **INF-γ** | Sham | RFA | -0.07 | -0.45, 0.3 | 0.7230 |
|  |  | Sham | CRA | -0.03 | -0.4, 0.36 | 0.8916 |
|  |  | RFA | CRA | 0.04 | -0.3, 0.38 | 0.8027 |
|  | **TNF-α** | Sham | RFA | -0.2 | -1.2, 0.8 | 0.6433 |
|  |  | Sham | CRA | -0.3 | -1.3, 0.7 | 0.5652 |
|  |  | RFA | CRA | -0.06 | -0.9, 0.8 | 0.8954 |
|  | **IL-1β** | Sham | RFA | 0.3 | -1.5, 2 | 0.7533 |
|  |  | Sham | CRA | 0.7 | -1, 2.5 | 0.4028 |
|  |  | RFA | CRA | 0.5 | -1, 2 | 0.5423 |
|  | **IL-2** | Sham | RFA | -0.004 | -0.5, 0.5 | 0.9873 |
|  |  | Sham | CRA | -0.05 | -0.6, 0.5 | 0.8522 |
|  |  | RFA | CRA | -0.04 | -0.5, 0.4 | 0.8478 |
|  | **IL-12p70** | **Sham** | **RFA** | **29** | **9, 48** | **0.0065** |
|  |  | Sham | CRA | 17 | -3, 37 | 0.0884 |
|  |  | RFA | CRA | -12 | -30, 6 | 0.1888 |
|  | **IL-5** | Sham | RFA | 2 | -2.9, 6.9 | 0.4068 |
|  |  | Sham | CRA | 2.8 | -2, 7.7 | 0.2464 |
|  |  | RFA | CRA | 0.8 | -3.4, 5 | 0.6958 |
|  | **IL-6** | Sham | RFA | 31 | -5, 67 | 0.0892 |
|  |  | Sham | CRA | 21 | -15, 57 | 0.2368 |
|  |  | RFA | CRA | -10 | -40, 21 | 0.5239 |
|  | **IL-4** | Sham | RFA | 0.03 | -0.1, 0.16 | 0.6373 |
|  |  | Sham | CRA | 0.07 | -0.05, 0.2 | 0.2552 |
|  |  | RFA | CRA | 0.04 | -0.07, 0.16 | 0.4427 |
|  | **KC GRO** | Sham | RFA | 5 | -65, 74 | 0.8871 |
|  |  | Sham | CRA | -40 | -109, 30 | 0.2545 |
|  |  | RFA | CRA | -44 | -104, 16 | 0.1413 |
|  | **IL-10** | Sham | RFA | -0.4 | -2, 1 | 0.5748 |
|  |  | Sham | CRA | -0.2 | -1.7, 1.3 | 0.7952 |
|  |  | RFA | CRA | 0.2 | -1, 1.5 | 0.7259 |

*Differences of least-squares means comparing different pairs of Treatment groups per cytokine and time point, based on the repeated-measures model. (P-values are unadjusted for multiple tests, and therefore only p-values < 0.016 (≈ 0.05/3 tests) should be considered as reflecting evidence of a difference.

Table S3. Cytokine level differences between time points.

| **Cytokine** | **Treatment** | **Estimate**  **6-72h** | **95% CI**  **(lower, upper)** | **P value*** |
| --- | --- | --- | --- | --- |
| **INF-γ** | Sham | -0.2 | -0.5, 0.1 | 0.1762 |
|  | RFA | -0.14 | -0.4, 0.1 | 0.2369 |
|  | CRA | -0.3 | -0.5, -0.04 | 0.0225 |
| **TNF-α** | Sham | 0.4 | -1.6, 2.5 | 0.6547 |
|  | **RFA** | **5** | **3.4, 6.5** | **<.0001** |
|  | **CRA** | **2.4** | **0.9, 4** | **0.0033** |
| **IL-1β** | **Sham** | **-2.5** | **-3.7, 1.3** | **0.0001** |
|  | **RFA** | **-2.4** | **-3.25, -1.5** | **<.0001** |
|  | CRA | -0.44 | -1.3, 0.44 | 0.3157 |
| **IL-2** | Sham | -0.14 | -0.8, 0.5 | 0.6875 |
|  | RFA | 0.3 | -0.2, 0.9 | 0.2240 |
|  | CRA | 0.35 | -0.2, 0.9 | 0.1991 |
| **IL-12p70** | Sham | -12 | -43, 19 | 0.4429 |
|  | RFA | -22 | -46, 2 | 0.0756 |
|  | CRA | -8 | -32, 16 | 0.5033 |
| **IL-5** | Sham | -1 | -5.4, 3.3 | 0.6388 |
|  | **RFA** | **-4.5** | **-8, -1** | **0.0106** |
|  | **CRA** | **-5.3** | **-8.6, -2** | **0.0033** |
| **IL-6** | Sham | 24 | -369, 417 | 0.9015 |
|  | RFA | 231 | -73, 536 | 0.1307 |
|  | **CRA** | **821** | **517, 1126** | **<.0001** |
| **IL-4** | Sham | -0.03 | -0.16, 0.1 | 0.6722 |
|  | RFA | 0.026 | -0.08, 0.13 | 0.6171 |
|  | CRA | -0.1 | -0.2, -0.005 | 0.0407 |
| **KC GRO** | Sham | 106 | -154, 366 | 0.4081 |
|  | **RFA** | **423** | **222, 623** | **0.0002** |
|  | **CRA** | **701** | **501, 900** | **<.0001** |
| **IL-10** | Sham | -3 | -9.3, 3.3 | 0.3337 |
|  | **RFA** | **13.2** | **8.3, 18** | **<.0001** |
|  | **CRA** | **6.3** | **1.5, 11** | **0.0133** |

*Differences of least-squares means comparing cytokine level at two time points per cytokine and treatment group, based on the repeated-measures model. (P-values are unadjusted for multiple tests, and therefore only p-values < 0.016 (≈ 0.05/3 tests) should be considered as reflecting evidence of a difference.


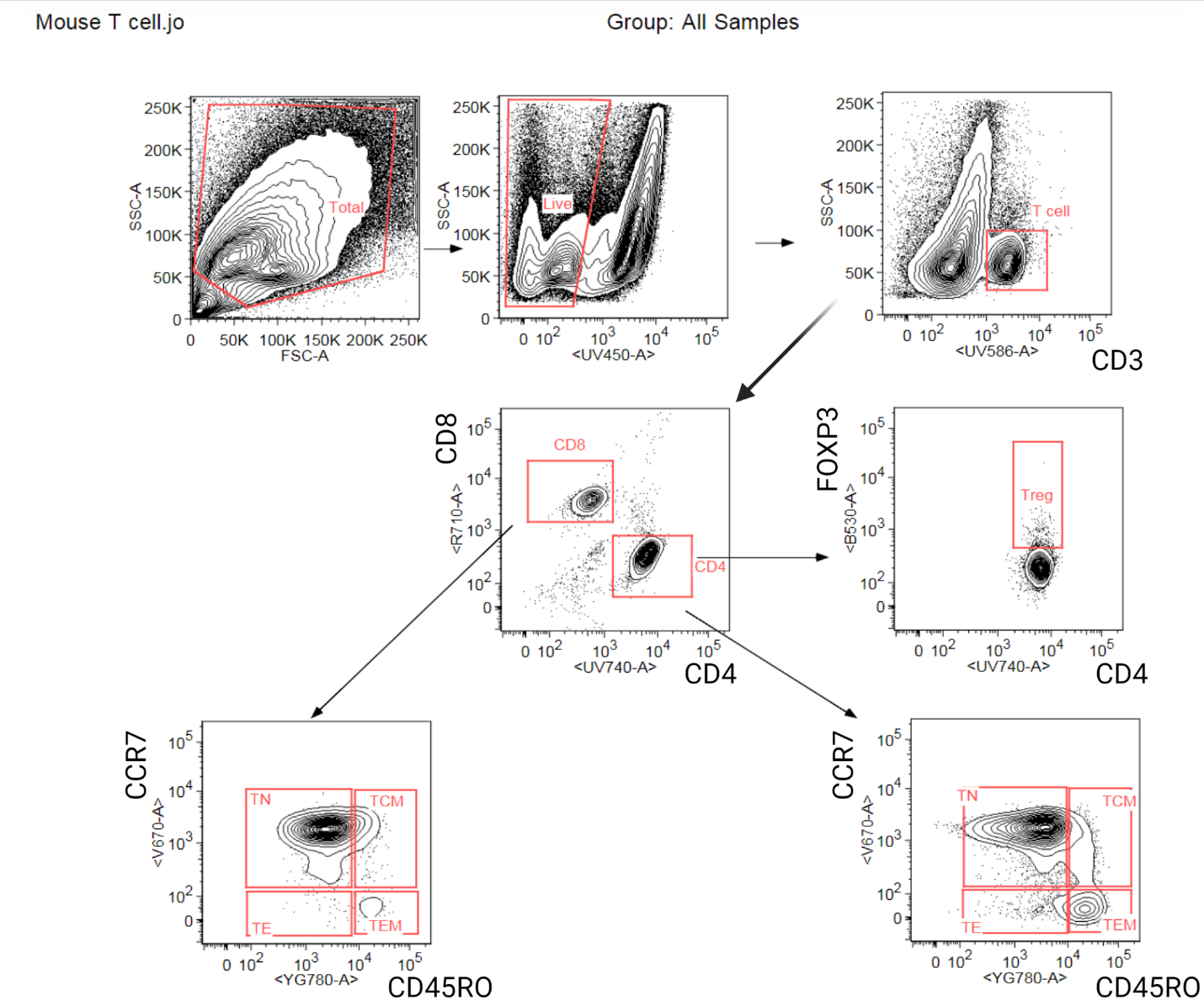


Figure S1.

Flow cytometry data and gating schemes for T cell population: first only live cells were gated, next T cells population was gated using CD3 marker, from this population various T cell sub-populations were determined using the markers CD4, CD8, FOXP3, CD45RO, CCR7.


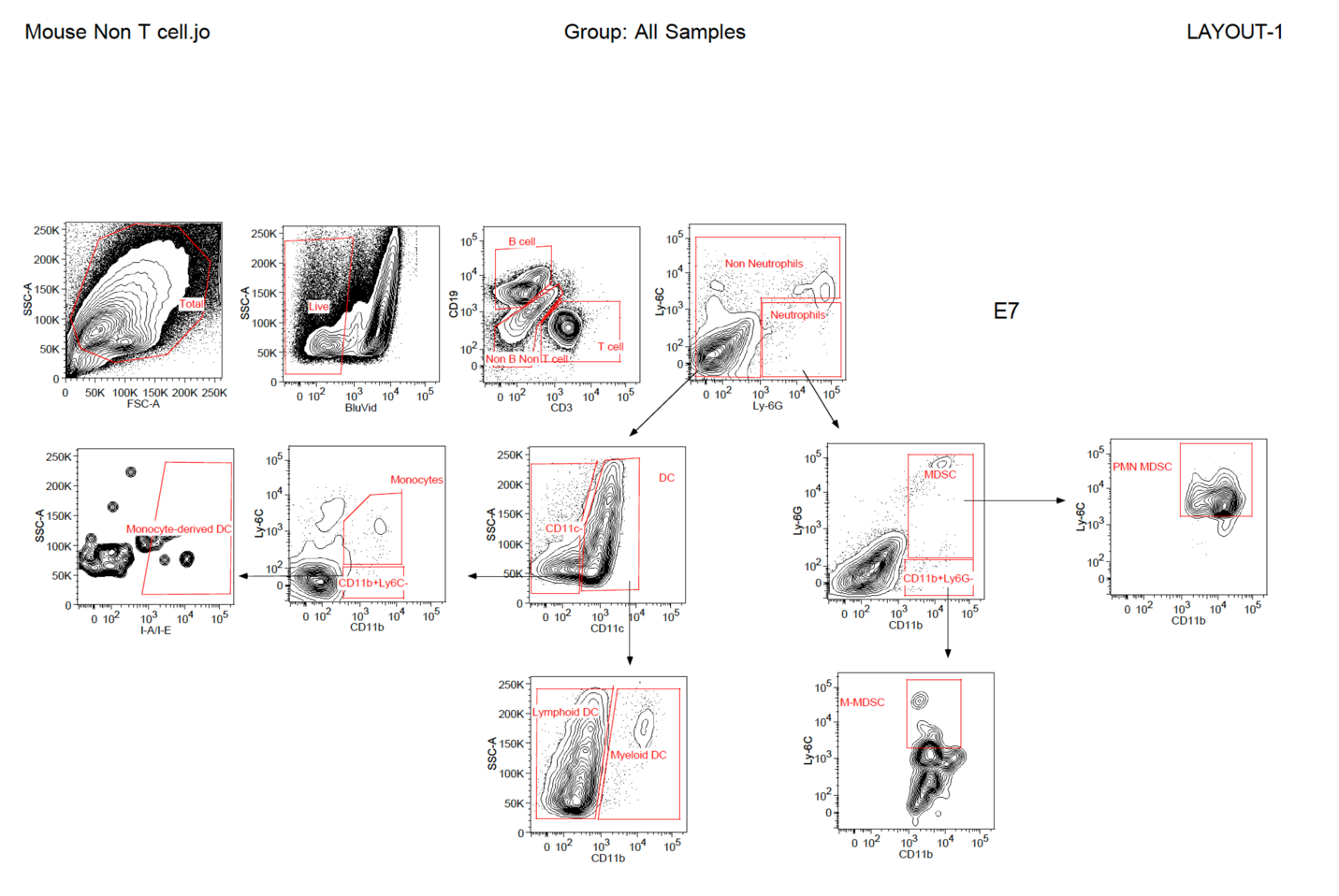


Figure S2.

Flow cytometry data and gating schemes for non-T cell population: first only live cells were gated, next non-T cells population was gated using CD3, and CD19 markers. Cell population negative to CD3 was subdivided into sub-populations using the markers Ly-6G, Ly-6C, CD11b, and CD11c.
